# Late Neolithic phytolith and charcoal records of human activities and vegetation change in Shijiahe culture, Tanjialing site, China

**DOI:** 10.1371/journal.pone.0177287

**Published:** 2017-05-19

**Authors:** Xiao Hong Zhu, Bing Li, Chun Mei Ma, Cheng Zhu, Li Wu, Hui Liu

**Affiliations:** 1 College of Geographic and Oceanographic Sciences, Nanjing University, Nanjing, China; 2 College of Business, Jiangxi Normal University, Jiangxi, China; 3 College of Resources and Environment, Hebei Normal University, Shijiazhuang, China; 4 College of Territorial Resources and Tourism, Anhui Normal University, Wuhu, China; 5 State Key Laboratory of Loess and Quaternary Geology, Institute of Earth Environment, CAS, Xi’an, China; 6 Hubei Provincial Institute of Cultural Relic and Archaeology, Wuhan, China; Institute of Botany Chinese Academy of Sciences, CHINA

## Abstract

There is significant archaeological evidence marking the collapse of the Shijiahe culture in the middle reaches of the Yangtze River in China during the late Neolithic Period. However, the causes for this cultural collapse remain unclear. Our sedimentary records from a 3.3 m long profile and 76 phytolith and charcoal samples from the Tanjialing archaeological sites provide records of interactions between an ancient culture and vegetation change. During the early Shijiahe culture (c, 4850–4400 cal BP), the climate was warm and humid. Fire was intensively used to clear the vegetation. In the mid-period of the Shijiahe culture (c, 4400–4200 cal BP), the climate became slightly dry-cold and this was accompanied by decreasing water, leading to settlements. From c, 4200 cal BP, severe drought eroded the economic foundation of rice-cultivation. These conditions forced people to abandon the Shijiahe ancient city to find water in other regions, leading to the collapse of the Shijiahe culture.

## Introduction

The study of the evolution of human civilization and the interactions of humans with the environment elucidates the development of human society [1]. Consequently, understanding how past cultures interacted with their environments provides insight into long-term human customs, traditions, and agriculture [2–3].

The Holocene epoch (c, 11000 cal BP-present) is the time period after the last glacial melt of the Quaternary period. “Holocene Event 3” occurred over most of the planet around 4000 cal BP [4]. This phenomenon included abrupt climatic variations and has been verified in the Alps in Europe (Perry, 2000), the North Atlantic region [4–6], West Asia [7–10], and the Peruvian mountains in South America [11]. “Holocene Event 3” is considered the coldest and most influential climatic event after the Younger Dryas [12], and this time period marks the end of the Climatic Optimum Period and the beginning of the late Holocene [13].

In China, much evidence supports the cooling trend of climate change, including monsoon effects in 4000 cal BP. Evidence for this change comes from several studies, such as δ^18^O analysis of the Dunde ice core [14], high resolution lacustrine sediment record in South China [15], glacial activity in Western China [16], pollen research in Inner Mongolia [17], foraminifera records of Okinawa trough [18], and marine sediment records in the South China Sea [19]. One of the most important turning points in human evolution history took place around c, 4000 cal BP in China. Many cultures, such as the Qijialing Culture (c, 5000–4600 cal BP) in West-North China, the Laohusha Culture (c, 5000–4000 cal BP) in Inner Mongolia; the Liangzhu culture (c, 5300–4500 cal BP), the Shijiahe culture (c, 4600–4000 cal BP) in the middle and lower reaches of the Yangtze River in Southern China, and the Longshan culture (c, 4600–4000 cal BP) in the lower reaches of the Yellow River, collapsed during this time period. Therefore, “Holocene Event 3” had important consequences for Chinese cultural change. Cultural recession can result from internal or external causes. Internal causes include politics, economy, war, and the development of society and culture [5, 20], and external causes are mostly environmental changes. Both human and environmental factors can have individual effects but can act synergistically under certain conditions. Our aim in this study was to combine results from the excavation site at the Tanjialing site with previous studies to provide new evidence to explain the cultural decline of the Shijiahe culture in the middle reaches of the Yangtze River.

**Fig 1:**
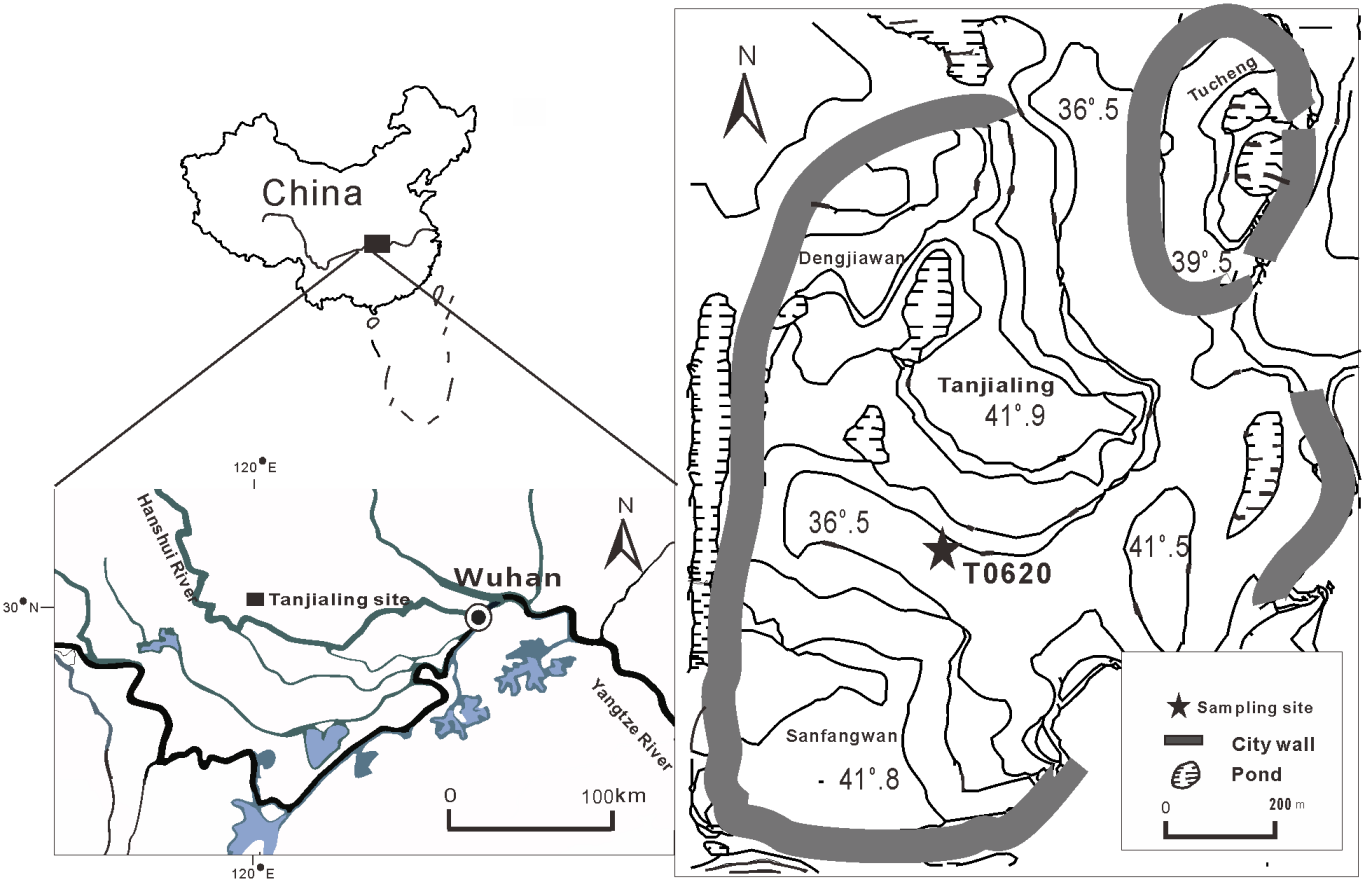
Geographical location of the Tanjialing site.

Abundant archaeological excavations in the Jianghan Plain have provided evidence for flourishing Neolithic cultures in the middle reaches of the Yangtze River (Fig 1). There were four successive Neolithic cultures that have been recognized: Chengbeixi culture (c, 8000–6300 cal BP), Daxi culture (c, 6300–5000 cal BP), Qujialing culture (c, 5000–4600 cal. BP), and Shijiahe culture (c, 4600–4000 cal BP) [21–22]. The Shijiahe ancient city was founded during the Qujialing and Shijiahe cultures, which were the most advanced cultural stages of these Neolithic periods [23]. This was the largest prehistoric city in China at that time, and is thought to have had 22000–30000 inhabitants. The perimeter of this city was 1200 meters long, 1100 meters wide, and covered an area of about 1.3 million m^2^. The ancient people also built a moat to protect the city, which measured approximately 4800 meters long by 80–100 meters wide by 6–8 meters deep in most places outside the rampart. Archaeologists thoroughly investigated this area, excavating more than ten sites including Tanjialing, located in the central region of this city (Fig 1) with a residential area of about 8km^2^. After lasting 1000 years, however, Shijiahe was abandoned, until the XiZhou Dynasty settled there about 3000BP [24].

The influence of environmental catastrophe was apparent in the mid-reaches of the Yangtze River [25–26]. The excessive development of the land by humans and climate drought caused the decline of the Shijiahe culture [27–28]. Wars resulted in the loss of resources, which further contributed to the recession of Shijiahe culture [29]. The end of the Shijiahe culture corresponded with a period of climate deterioration and monsoon weakening, resulting in long-term drought [30]. Other studies indicated that there were significant flood events [31] and climate change during this time period [27]. Despite intensive palaeo-environmental research and associated archaeological studies over the last 30 years, our understanding of the decline of this once flourishing ancient city remains incomplete.

Our research group implemented sampling at Tanjialing site in 2011 after archaeologists’ excavation. The organic rich sediments were organized chronologically and vegetation change was demonstrated by microfossil analysis. We previously published results of pollen measurement and identification from this site, providing a broad perspective on the interactions between human activity and environmental changes [32].

To expand on that work, further investigation was performed on phytoliths. The phytoliths have high stability and resistance against decomposition and weathering, allowing them to persist for long periods of time in soils, thus forming a record of past vegetation. Phytoliths have also excellent high temperature resistance [33–35]. These advantages allow phytolith records to be considered as precise, stable, and reliable sources of archaeological information.

## Regional setting

A trench was excavated at Tanjialing site (30°46′17.39″N, 113°04′48.27″E), located about 100 km northwest of Wuhan City in the Jianghan Plain, Hubei Province. This site lies in a shallow depression that is today largely used for intensive paddy rice production, with altitudes that are 33 m above mean sea level (AMSL). This area is typically dominated by a monsoonal climate with a mean annual rainfall of 1000–1300 mm and floods from June to August. The mean annual temperature is 16°C and the winter temperature is 3.5°C cooler. The lakes and wetlands are densely developed.

**Fig 2:**
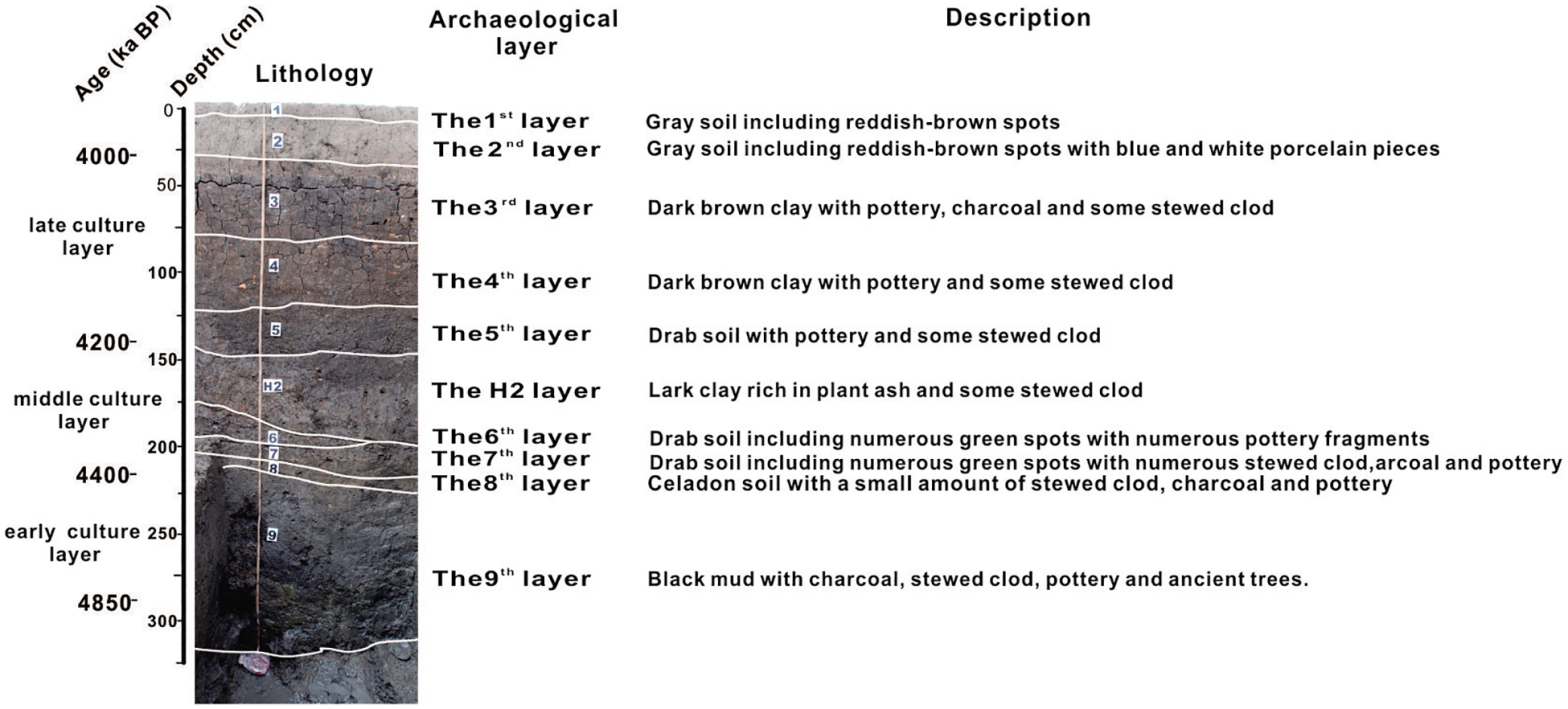
Lithology, archaeological stratigraphy, and description of the sampled profile, T0620, at the Tanjialing site.

The vegetation in the Shijiahe region is subtropical deciduous and evergreen broadleaved mixed forest (Fig 2), but the little amount of forest that remains today is dispersed, due to human activities. The plants present at higher elevations in this region are *Pinus massoniana* forest, secondary shrubs, and aquatic vegetation. On the mountain, the canopy is dominated by trees such as *Pinus massoniana*, *Quercus variabilis*, *Quercus glandulifera*, *Castanopsis sclerophylla*, and *Cyclobalanopsis* [27]. Vegetation in the foothills near Shijiahe city consists of *Quercus albus*, *Quercus glandulifera*, and shrubs. These regions have been converted to fields primarily for the cultivation of rice, wheat, and cotton.

## Methods

Our study did not involve human participants, specimens, or tissue samples, or vertebrate animals, embryos, or tissues.

Endangered or protected species were not included in these field studies.

The location of the field studies was a site that was previously used by researchers from the Hubei Provincial Institute of Cultural Relic and Archaeology. This Institute gave us the permission to further study this already excavated location.

### Field work

The excavation in the area of Tanjialing site was performed in two stages, first in 1982 and then again in 2011. During the South-to-North Water Diversion, a site rescue operation by the Hubei Provincial Institute of Cultural Relic and Archaeology preserved the Late Neolithic Site. A total area of 200 m^2^ was excavated.

The wall of the trench was cleaned by steel shovel to reveal a fresh profile, and integral cutting of archaeological strata was performed according to a box column sampling method, where samples were put in 4 stainless steel, 100 cm long containers [36]. After sealing, samples were transported to the laboratory for further preparation, weighing, and sub-sampling. This trench represents an optimal stratigraphic sequence with a thickness of 330 cm and was divided in nine lithological layers composed of three distinctive occupation sections in the south wall of trench T0620 (Fig 2). A total of 76 samples, each 20 g dried powder sample was collected from 8 cm to 330 cm depth, and then was crushed and air-dried for laboratory analysis. The precise location of the samples and the sequence in which they were removed was recorded before sampling to prevent any mixture between layers. Samples were taken continuously from the freshly cleaned exposed profile at 5 cm intervals within a depth range of 8–215 cm and at 2 cm intervals for the depth range between 215–330 cm.

### Experiment

#### Radiocarbon dating

Two samples (T0620-H2 and T0620-9) were submitted for AMS C-14 dating. Sample preparation was performed at the Chinese Academy of Sciences, Guangzhou Geochemistry Institute, and the prepared samples were then dated by the Nuclear Physics and Nuclear Technology State Key Laboratory of Peking University and calibrated using CALIB 6.0.1 software [37].

#### Phytolith analysis

Sample preparation for phytolith and micro-charcoal analysis was performed using a procedure slightly modified from Piperno [38] and Runge [39]. Basically, concentrated 30% hydrogen peroxide (H_2_O_2_) solution was used to remove the soil organic matter while keeping the temperature constant at 70°C in a water bath. Next, 15% hydrochloric acid (HCl) was added to dissolve the calcareous minerals. The solid clay particles were separated from pollen, phytolith, and charcoal by adding zinc bromide (ZnBr_2_) solution. The liquid phase was removed using a Pasteur pipette and then was centrifuged to obtain the solid samples containing the concentrated phytolith and charcoal material. This solid mixture was homogenized with glycerol and transferred to a microscope slide. *Lycopodium* spores were added as markers prior to processing to estimate phytolith and micro-charcoal concentrations and influx rates. Phytolith residues were mounted in silicon oil and scanned under an Olympus Nikon E200 microscope at 400X magnification. More than 250 phytolith grains were counted for each sample type. Phytolith abundance was expressed as percentages of all phytoliths counted. Identification was aided by the use of reference materials [40–44] and published guides [38, 39, 43, 45, 46]. The phytolith types identified in this research and their botanical affinities match those reported by Itzstein-Davey (2007a) [47]. Rice phytoliths typically divide into three morphotypes: fan-shaped, bilobate (dumbbell), and double-peaked glumes [48–53].

In this study, phytoliths were divided into 20 types according to the classification system of Lu [42], Twiss [44], and Wang [54], as follows: (1) arboreal gymnosperm types and broad-leaf-type); (2) dumbbell; (3) cross; (4) long saddle; (5) short saddle; (6) fan-rice; (7) double-peak (8) dumbbell-rice (9) phragmites; (10) trapezoid; (11) fan-shaped; (12) square; (13) rectangle; (14) stick-elongate; (15) sinuate-elongate; (16) smooth-elongate; (17) point; (18) rondel; (19) cylindric sulcate and (20) truncated-cones.

Wang [54] used the previous classification by Lu [42] to establish the Warmth index (Iw), which is a reference to the temperature variation in time. The Warmth index (Iw) is proposed to be ratios of the abundance of the warm-type grass phytoliths to the total amount of the warm- and cool-type grass phytoliths (rice phytoliths excluded), to reconstruct the temperature at the time of the samples. Festucoid, elongate, and acicular compose the cold types, while Chlordoideae, Panicoideae, cuneiform (including square and rectangle), and concave saddle belong to warm types.

**Fig 3:**
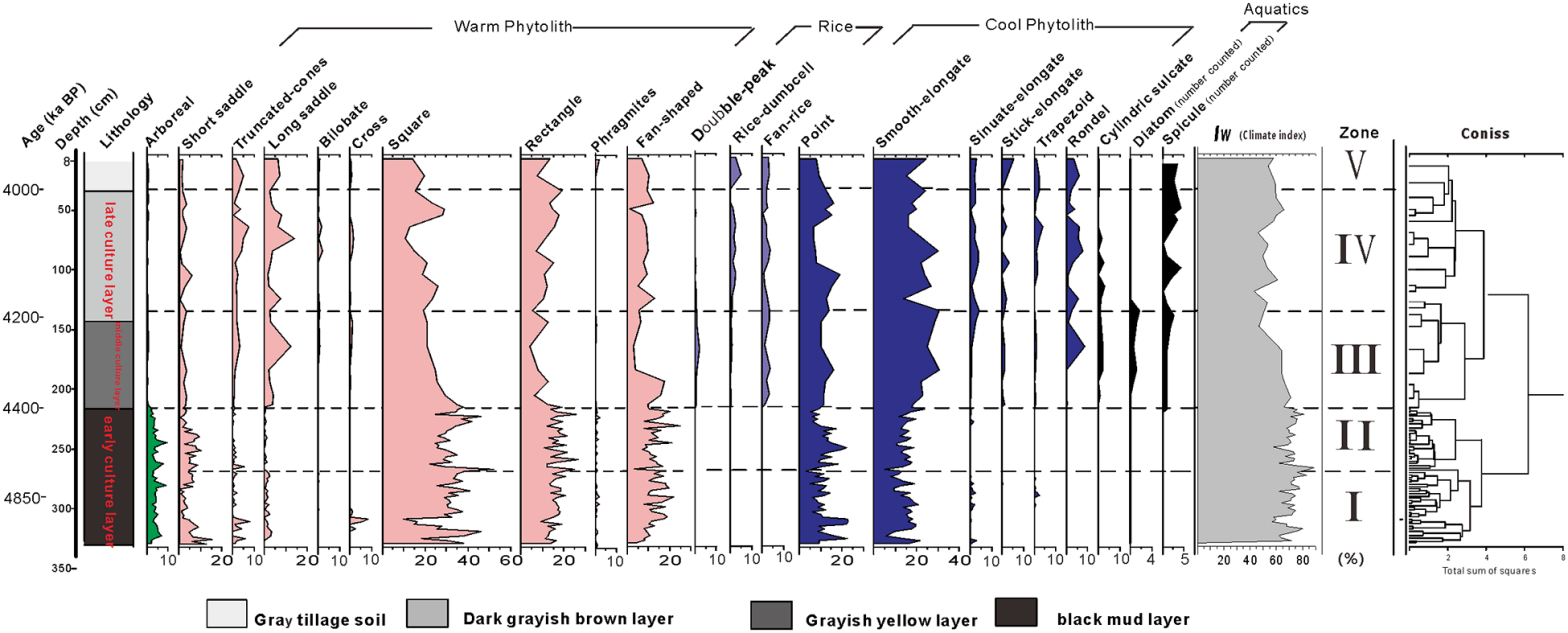
Phytolith percentage diagram for Tanjialing site.

**Fig 4:**
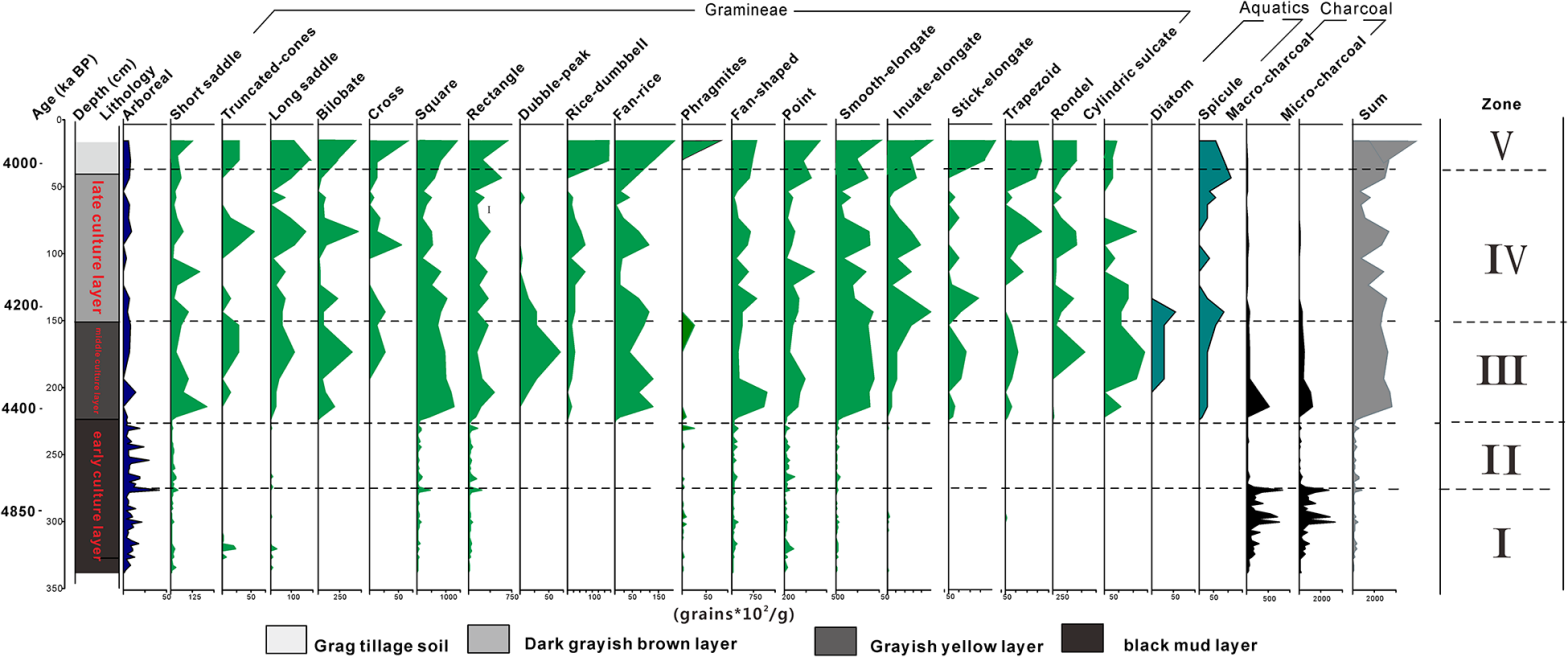
Phytolith concentration diagram for Tanjialing site.

Diagrams summarizing the sediment-based data from the site (Figs 2–4) were prepared using Tilia 2.0 software and the zonation was corroborated by the result of CONISS in TG view 2.0 [55–56]. The phytolith results are presented as percentages of the total classified phytolith sum and included a minimum of 250 single-celled morphological types that were counted at each sampled level.

#### Charcoal analysis

Charcoal and phytolith particles were counted under a microscope and compared to the number of the Lycopodium spores used as an added standard. Charcoal particles were divided into two size classes of 50–100 μm (micro-charcoal) and 100 μm and above (macro-charcoal) in diameter. Particles less than 50 μm in size were not included in the analysis, because they may have arisen from the fragmentation of particles during sample preparation. Diatom and spicule counts are shown as numbers counted throughout the profile because of their rare small amounts. Rice-type phytolith counts were generated, but they were not separated into likely wild and domestic varieties. Full palynology analyses were completed and published elsewhere [32] and were not repeated here.

All the species were counted by using a Nikon microscope E200 at a 400-fold magnification and reported as concentrations (particles/g).

## Results

### Stratigraphy

Within this depression, the unconsolidated sediment sequence comprises a 1–1.5 m thick black clay layer at the base, coarsening upwards to incorporate sands and gravels, and then topped by a layer of brown organic-rich silty clay (Fig 2). These layers contain highly organic clay material, abundant plant remains, and naturally deposited organic debris that may include archaeological material. They are sealed by a layer of grey clay up to a half-meter thick that becomes laminated in its upper part and forms a mixed topsoil at the surface.

### Time sequence

AMS C-14 dates obtained for this study are shown in Table 1. The Shijiahe culture is divided into three periods: early (9th archaeological layer), middle (8th-H2 archaeological layers), and late culture periods (5th-3rd archaeological layers) [24]. Chinese scholars have published numerous chronological studies of Shijiahe culture [57–59]. We used the chronological data from Mao [60] for reference because Mao and our group excavated samples from the same southern trench wall (T0620). Mao placed the Shijiahe culture between c, 4850 cal BP (280cm, the 9^th^ archaeological layer) and c, 4124 cal BP (35cm, the 2^nd^ archaeological layer). Our research focus was the determination of the layer age of the middle (8^th^-H2 archaeological) layer, and two of our samples were sent for AMS C-14 dating. The identified ages were 4356, and 4338 cal. BP for the two samples. Combining the dates of the samples from our group and those of Mao’s, we deduce that the age of the H2 to 8th layers at the Tanjialing site is about 4400–4200 cal BP, which is attributed to the middle Shijiahe culture. Late Shijiahe culture is approximately in the range between c, 4200–4000 cal BP according to the age of the 3rd to 5th archaeological layers. The age of the early archaeological culture (9^th^ archaeological layer) is c, 4850–4400 cal BP (Table 1).

**Table 1:** AMS ^14^C dating and its calibrated ages of the excavation trench T0620 in the Tanjialing Neolithic site.

Sample	Material	Lab NO.	Depth	Calibrated ^14^C age /1δ BC	Calibrated ^14^C age /2δBC	Medium age (cal. aBP±1δ)	Medium age (cal.aBP±2δ)	
T0620-H2	Charcoal	GZ5043	180cm	2470 (42.54%) 2434	2475 (96.55%) 2336	4402±18	4356±70	This study
T0620-9	Charcoal	GZ5044	218cm	2457(52.04%) 2418	2407(88.08%) 2376	4388 ±18	4342±16	This study
TJL-2^14^ C-3	Charcoal	Unclear in the orginal reference	35cm			3769±39	4124±38	(Mao,et al,.2014)
TJL-2^14^ C-1	Charcoal	Unclear in the orginal reference	195cm			3977±40	4432±16	(Mao,et al,.2014)
TJL-1^14^ C-wood	Wood	Unclear in the orginal reference	280cm			4284±41	4850±23	(Mao,et al,.2014)

### Phytolith data

**Fig 5:**
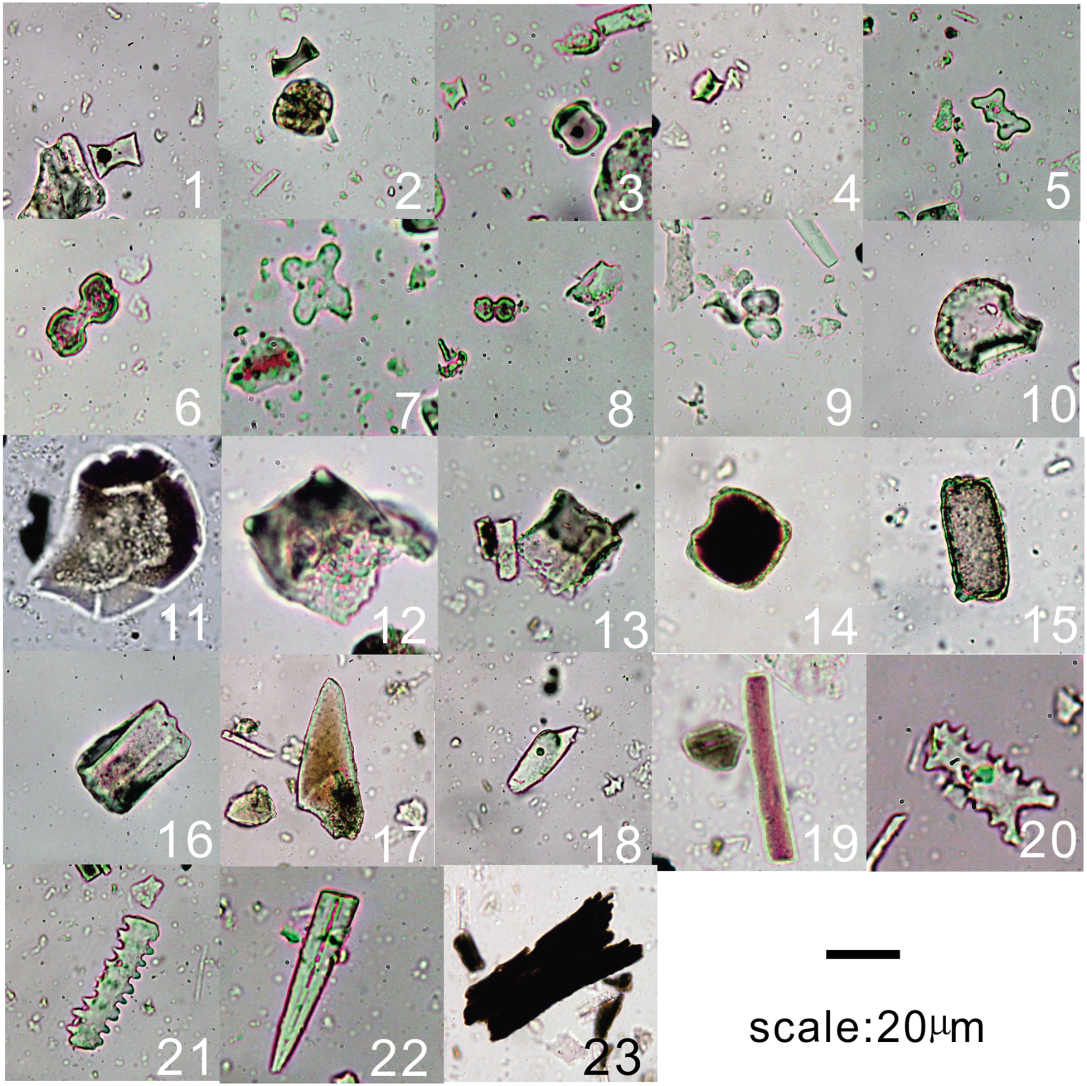
Pictures of phytolith (1st-21st), spicule (22nd) and charcoal (23rd) morphotypes identified at the Tanjialing site. (1–2) long saddle; (3) short saddle; (4) rondel; (5–6) dumbbell; (7) cross; (8–9) rice-dumbbell; (10) fan-rice; (11) fan-shaped; (12) double-peak; (13–14) square; (15–16) rectangle; (17–18) point; (19) smooth-elongate; (20–21) stick-elongate; (22) spicule; and (23) charcoal.

All phytolith and charcoal data from Tanjialing are presented in Figs 3 and 4, and the morphotypes are presented in Fig 5. These figures show the five main sedimentary zones were identified, indicating major changes in the depositional environment and/or archaeological context as described below.

Zone I (330–268 cm): The phytolith concentration was very low (in the range of 3100–23900 grains/g, mean 11800 grains/g; Fig 4). Though grasses are dominant, the arboreal taxa are maximum (1.9%-7.0%) in this zone. Warm-type phytoliths such as square (9.9%-51.9%), rectangle (9.2%-24.7%), and fan-shaped (4.3%-21.7%) are abundant and Phragmites are present at a low percentage (0.4%-1.8%). Cold-type phytoliths, which are represented mainly by elongate (4.9%-21.5%), and point-shaped (3.3%-22.3%) forms, are at much lower abundance than the warm-type phytoliths. Stick-elongate and rondel are absent in the cold-type category. There are multiple short cell forms, with five classes represented: rondel, sinuate, bilobate, short saddle, and long saddle. Short saddle (2.4%-15.6%) and long saddle (0.4%-8.1%) are well represented in the early stage of the lithological regime. These forms correspond to the Choridoid and Bambusoid sub-family, the important C_3_ grass group that is capable of growing in warm areas. Rondel and trapezoid forms, linked to the Pooideae sub-family, were not identified. The Warm index (Iw) varied from 0.6 to 0.89 and the mean value was 0.75.

Zone II (268–310 cm): The phytolith concentration ranged between 10900–88900 grains/g (mean 24100 grains/g; Fig 4). The assemblages of phytoliths were less diversified than those of zone I.

Among the long cells, there were several from the warm type group such as square (24.6%-37.9%), rectangle (10.6%-25.5%), and fan-shaped (9.3%-24.8%). The cold type group included point-shaped (6.4%-22%) and elongates (10.4%-23.6%), with increased frequencies in the upper part of the section. Of the short cells group, there were more bilobate (0.3%-1.1%) and short saddle (0.4%-9.4%) phytoliths than in the previous zone. Rondel and trapezoid were in zone II. The arboreal (1.3%-7.5%) class was represented by Dicotyledonous taxa, the same as in zone I. The Iw ratios varied from 0.61 to 0.72 with a mean value of 0.66.

Zone III (110–218 cm): The phytolith concentration in this zone was in the range of 93500 and 363400 grains/g (278800 grains/g mean; Fig 4), almost ten times higher than in Zones I and II. The dominant frequencies (Fig 3) were rondel (0.3%-8.3%), sinuate (0.3%-0.8%), and bilobate (1.8%-12%) phytoliths. These results indicate that Pooideae and Panicoideae sub-families coexisted in Tanjialing, suggesting a shift in edaphic or vegetable conditions. This zone also contained Poaceae assemblages, similar to that of the previous zone. However, both the relative abundance and influx of the arboreal species were reduced (0.3%-0.8%). Further, there was a significant decline in the square (20.8%-34.9%), rectangle (4.1%-21.1%), fan-shaped (2.9%-17.5%), and long saddle (0.4%-3.3%) forms, and an increase in the smooth-elongate (17.9%-30.5%), stick-elongate (0.3%-1.2%), and rondel (0.3%-0.8%) forms, suggesting that cold-dry environments may have existed at the sampling site. In this same zone, the presence of double-peak (0.3%-2.0%), rice-dumbbell (0.3%-0.8%), and rice-fan (1.2%-3.7%) phytoliths and the disappearance of *Phragmites* suggest the use of land for agricultural purposes. There are spicules, but their numbers are few (values in the single digits), extending from this zone to zone V. The Iw ratios varied from 0.45 to 0.65, with a mean value of 0.52.

Zone IV: The phytolith assemblages (Fig 3) in this zone showed a decrease in square (10.7%-26%), rectangle (5.2%-18.8%), and fan-shaped (1.5%-12.9%) forms, but an increase in long saddle (0.9%-7.7%), double-peak (0%-0.9%), rice-dumbbell (0%-4.8%), rice-fan (0.4%-3.6%), rondel (0.9%-6.1%), and stick-elongate (0%-2.1%) forms. The phytolith concentration fluctuated to twice as high as before and the lowest value was close to that of c, 4000 cal BP, with generally high levels throughout this zone in the range of 65200 and 334400 grains/g (mean of 193700 grains/g mean) (Fig 4). The Iw ratios varied from 0.44 to 0.62 and the mean value was 0.49, the lowest value of the entire profile.

Zone V: At the top of the soil and nearby, the counted phytolith numbers represent an average because the intensive mixing of the soil during plant cultivation. These numbers indicate a dramatically improved effectiveness of rice cultivation over time. A large number of rice-type phytoliths were obtained from a modern paddy, and used for comparison for the older samples.

### Charcoal

The up-profile variations in the micro-charcoal appear to correlate with the age zones (Fig 4).

The concentration profiles for micro- and macro charcoal showed the same trend of change the magnitude of numbers. Zone I and III showed a significant amount of counted charcoal residue compared to Zone II and Zone VI-V.

In Zone I, cal. 4800–4600 BP, the mean concentration value was the highest of the profile, the macro-charcoal was 25500 grains/g mean, and micro-charcoal was 114000 grains/g mean. The highest peaks of the micro-charcoal and macro-charcoal concentrations were 82900 grains/g (268 cm) and 256000 grains/g (285cm), respectively.

At the top boundary with Zone I, the concentrations in Zone II cal. 4600–4400 BP, macro-charcoal (1500 grains/g mean), and micro-charcoal (9100 grains/g mean) were much lower and showed little change through the Zone II.

In Zone III, cal, 4400–4200 BP, the concentrations for both the macro-charcoal (20300 grains/g mean) and micro-charcoal (70800 grains/g mean) were highest.

Both phytolith Zone IV and V showed very low macro-charcoal (1300 grains/g mean) and micro-charcoal (5300 grains/g mean) concentrations with even less variability compared with the other zones.

The charcoal concentrations showed considerable variations. The largest macro-charcoal peak occurred in Zone I (292 cm), but unlike the micro-charcoal, the boundary was not marked by the highest macro-charcoal concentration although these boundaries remained consistent at other zones.

Also, the presence of diatoms and sponge spicules were noted in Zone III, but not the other zones.

## Discussion

The Jianghan Plain developed by flooding the Yangtze River and Hanjiang River catchment areas. Tanjialing, situated in the northern Jianghan Plain, is located near the boundary between the subtropical and medium latitude monsoon climates. This site is rich in artifacts and biological remains, and thus is ideal for tracing the late Neolithic human impact on the local landscape.

### Response to climate changes

In northern China, the environmental effect of cooling events (Holocene Event 3) resulted in drought. The study of the Hai Dai inland lake, which is located at the edge of the east Asian monsoon region, showed that the water level was maintained at a high level from 9500 to 4000 cal BP. Next, the lake shrunk sharply, around 4000 cal BP [61–62]. A pollen analysis profile from peatland showed that the climate was dry around 4100 cal BP on the Tumb Plain in Inner Mongolia in the marginal area of the East Asian monsoon area [63]. The study of Yiema Lake sediments in the arid desert region demonstrated that the summer monsoon became weak around 4100 cal BP [64]. However, in southern China, some scholars believe that the environmental effect of a large-scale cooling event may have caused an increase in precipitation [65]. The research in the Mian Yan Plain (in the middle reach of Yangtze River) indicated that the Holocene thermal maximum occurred during c, 6700–4400 cal BP. This maximum followed a transition and from c, 3900–1700 cal BP, the temperature decreased, but the humidity remained very high. The range of moisture content increased during the Holocene Optimum Period and the water level of lakes also rose [66]. The distribution of Neolithic cultural sites in Jianghan Plain, historical data, and buried ancient trees illustrate the frequent flooding that occurred during the period of 4700–3500 cal BP [25].

**Fig 6:**
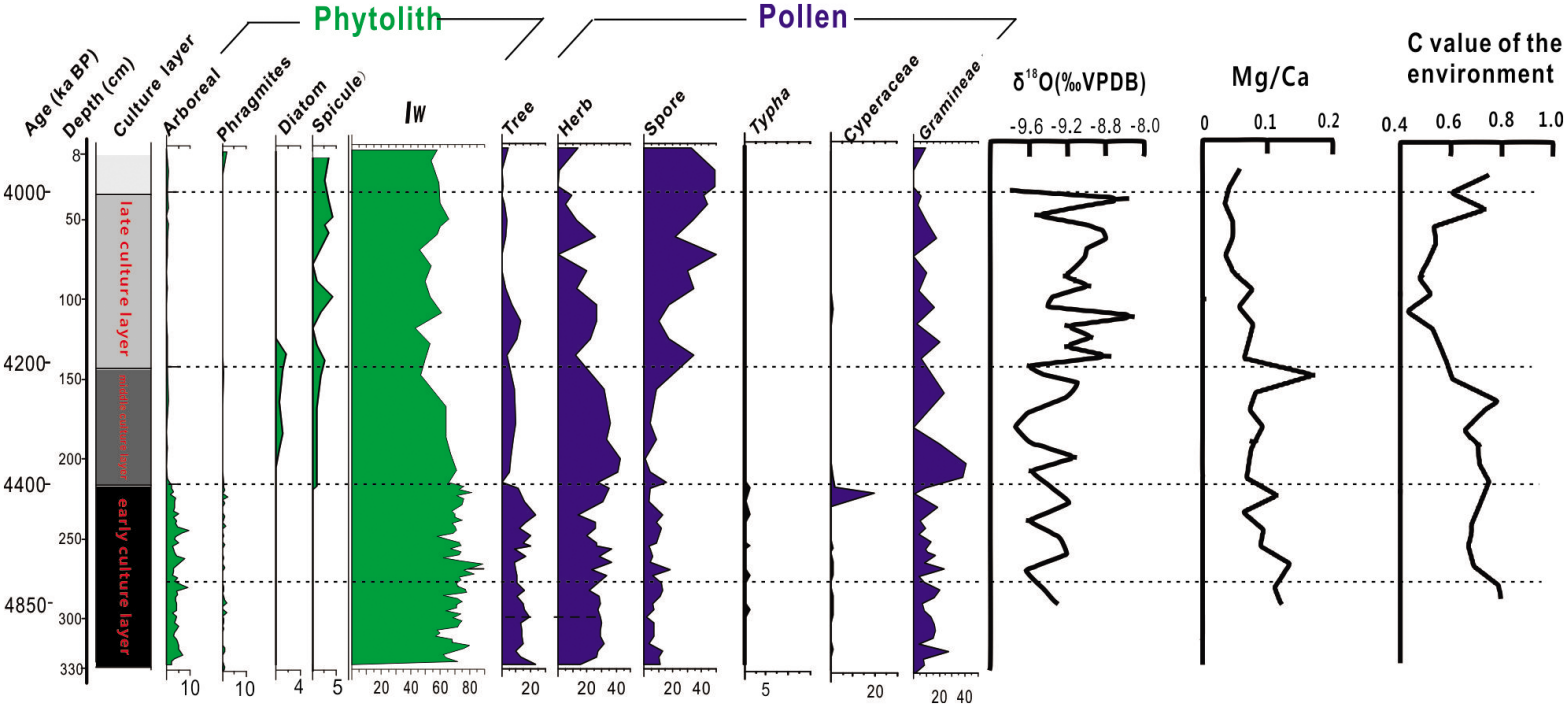
Phytolith (this study), pollen [32], Mg/Ca, and C value of the environment [60] of sediment-based data in Tanjialing T0620, as well as the record of δ^18^O for Hs-4 [71].

An important question remains about potential environmental effects of the Eastern Asian monsoon area on the climate during this time period. Generally, a cooling climate is associated with drought, but the environmental effects are not consistent. In the North Monsoon region, the cooling events manifested mainly as drought, but in the South Monsoon region, the cooling events were accompanied with an increase in precipitation. Therefore, additional study is required to test and better understand these effects. Results from phytolith counting in this study indicated that between c, 4850 and 4400 cal BP, the local area was densely forested, as shown by the abundant levels of arboreal phytoliths including broadleaf-type, and Gymnosperms. Diverse squares, rectangles, and fan-shaped phytoliths and the high Iw value indicate warm and humid conditions in this phase. The presence of a fan-type phytolith is a major indicator of high moisture content in vegetation [67] (Fig 3). Presumably this area was subjected to the strengthened monsoon during early Shijiahe culture, producing a warm and wet environment. Artifacts recovered during the archaeological excavations included a large number of buried trees unearthed at the bottom of the 9th layer, and pottery describing an elephant may suggest that the appearance of the elephant’s image could be related to the tropical climate [68]. In addition, the ancient city wall/embankment was designed to protect the city against flooding at the Jianghan Plain during this phase (Zone I and II), as supported by the archaeological records and previous studies [26, 27, 32, 69,70]. The pollen evidence is similar to the phytolith data, suggesting a mixed evergreen and deciduous type of forest [27,32]. Moreover, the indices of larger Mg/Ca and C value of the environment indicate the climate was more humid and warm in this stage [60]The analysis of δ18O showed that the climate became dry-cold after c, 4300 cal BP [71] (Fig 6).

Water expansion was clearly a defining factor of hydrological history based on the evidence of Phytolith and lithology in this site. The water plants grew on low-lying lands, such as *Phragmites*. Natural and cultural organic sediments corresponding to early Shijiahe culture were intercalated within the thick deposits of black mud (215–330 cm). In addition to pollen, other evidence (Cyperaceae, *Typha*, and freshwater algae) was found and described by Li et al. (2013); they found no existence of diatoms and spicules because aquatic silicon species were not preserved in the presence of streaming water [38]. After c, 4400 cal BP, the vegetation fluctuation was primarily driven by climatic variations. This period may have been characterized by increasing levels of human activity. This conclusion came from the abundant charcoal and the marked shift in vegetation from forests to bush-herb-dominated open landscapes. The retreat of the water regime was much more pronounced in the Tanjialing (Zone III) where C_4_ and *Phragmites* decreased and were replaced by C_3_ grasses and the presence of Festucoid phytoliths, indicating a relatively dry and cool climate. C_3_ grasses and Festucoid phytoliths were represented by stick-elongate, trapezoid, and rondel shapes, which were absent before this phase. Also, warm-type phytolith numbers fell continually, especially fan-type, possibly suggesting a decrease in moisture and temperature (Fig 3). Pollen assemblages were dominated by herbs, which strongly indicates agricultural activities [32].

The archaeological profile records show a water regime retreat at the site due to the presence of stewed clod, charcoal, and pottery. These artifacts were excavated in the 8th layer, corresponding to the middle Shijiahe culture (4400–4200 cal BP) [68]. The water withdrawal suggests an effective decrease in wet conditions and indicates a cold and dry climate in mid-late Shijiahe culture, consistent with previous studies [27, 32, 60, 70].

### Human activity and cultural process

#### Fire regimes

Charcoal is one of the most important evidence for the reconstruction of fire history and ancient vegetation [72, 73]. Fire events can be related to biomass, climate, and sedimentary environment because charcoal is produced when vegetation is burned and is related to human activity.

Biomass and climate can affect the intensity of fire and its frequency [74]. Micro-charcoal carried by either the wind or water may have been deposited from distant trees and grasses after fires occurred. The macro-charcoal particles were obtained near the origin location of the plants [75–77].

In the early Qujialing—late Shijiahe culture (c, 4800–4500 cal BP), the macro-charcoal concentration was several times larger than that of the micro-charcoal. Maximum micro-charcoal concentrations were identified c, 4500 cal BP, and maximum macro-charcoal concentrations occurred at a later phase. These high macro-charcoal concentrations suggest an increase in human activities such as anthropogenic logging and burning plants. The clearance of the native vegetation was necessary to meet the demands for living place and for the farming of sufficient amounts of food to support the population of the ancient city. Evidence for fire regimes are the black and brown colors on the phytolith surface, indicating significant decolorization caused by the adsorption of carbon.

Charcoal concentrations decreased from c, 4500–4400 cal BP because land clearing exhausted natural resources. The subsequently increased charcoal concentrations after c, 4400 cal BP indicated that humans occupied land that was previously unsuitable for living and began to use fire to clear the forest and grassland. The appearance of rice phytoliths indicates active farming. The lower concentration of charcoal after c, 4200 cal BP in the lowest level may correspond to the reduction in vegetation biomass near Tanjialing site due to the dry-cold climate and when the people abandoned this region to find water.

#### Culture process

The Holocene Thermal Maximum increased rice agriculture yield steadily. This system of wetland rice farming in the Middle Yangtze dates from about c, 6300–5900 cal BP [78–79]. Archaeological evidence shows that rice was largely cultivated in the middle reaches of the Yangtze River c, 6500 cal BP by the Neolithic Daxi people [80]. Thickly layered carbonized rice husk and tools including stone celts, spades, froes, and hoes were found, suggesting advanced early agricultural activity. During the Qujialing culture, rice was the main food source in this area [80], and rice agriculture was an important driver of development within the Shijiahe culture (c, 4600–4200 cal BP). The flotation tests of the soil samples, from both the Tanjialing site and the Sanfangwan site, indicated that the main subsistence level was grain farming with minor wild plant resource utilization. Wild food supplies had clear superiority in the Tanjialing site, and grain was more advantageous at the Sanfangwan site [81]. Interestingly, rice phytoliths were not found in this stage (c, 5000–4400 cal BP, the 9th layer) suggesting running water conditions were not suitable for rice cultivation. The people likely cultivated the rice at higher elevation or outside of the ancient city.

The appearance of Oryzoideae phytoliths (double-peak, rice-dumbbell, and rice-fan shapes; Fig 3) and their abrupt increase in concentration after c, 4400 cal BP, indicates the cultivation of rice after the water retreated. The observed large amount of rice glume (double-peak type phytolith) suggests (165 cm, c, 4390 cal BP) that in layer H2 that there was a storage pit of rice. Pollen assemblages were dominated by herbaceous pollen such as Poaceae and *Artemisia* pollen, which strongly suggests agricultural activities as well [32]. Presumably, the ancient people may have developed irrigation techniques for the rice fields, because the climate became cold and dry, the lakes shrunk, and the underground water level dropped dramatically. Further, to maintain the social structure and the city population, the establishment of new agricultural fields was necessary.

Falling concentrations in phytolith samples, from c, 4200 to 4000 cal BP are consistent with vegetation degradation, particularly in the upper section of zone IV (45–150 cm). The chronic drought not only hindered local agriculture but also decreased the quality of the groundwater. The ancient humans were forced to migrate to lower altitudes to obtain water, limiting Shijiahe cultural development. Archaeological evidences of tombs during this period of the Shijiahe culture were unearthed at Sanfangwan site, at a lower elevation than that of the Tanjialing site [24].

Compared with a modern paddy (zone V), the rice concentrations in these sediments were quite low, suggesting that rice cultivation techniques were quite primitive during the Shijiahe culture stage (Fig 4). Additionally, the rice yield was vulnerable to seasonal and flash flooding. Rice is a vital component of a subsistence economy that includes other complementary survival strategies such as collecting, fishing, and hunting.

Many studies have reported that the reason for the decline of pre-historic civilization and national migration throughout low-latitude regions of the entire Northern Hemisphere was a drought event around 4200 cal BP. These effects were extremely intense in certain areas in China [15,82]. Alternatively, some researchers have proposed that Shijiahe cultural deterioration was caused by wars between the Sanmiao tribe and Huaxia tribe [83]. Our research supports the model that effects of climate change were contributing factors to this deterioration.

The climate changes caused fluctuations in lake sizes during the Qujialing and Shijiahe cultural periods (c, 5000–4000 cal BP), resulting in territorial adjustment of these sites [84–85]. The abandonment of the city during the late period of Shijiahe culture was due to the gradual disappearance of the protective water. All the urban sites were concentrated southeast of Shijiahe during this period. Chronic drought around 4200 cal BP not only hindered the development of local agriculture but also disrupted the irrigating ditch system. The ancient people who lived in this area were forced to migrate to lower altitudes to obtain water, leading to the stagnation of Shijiahe cultural development.

## Conclusions

Phytolith and charcoal investigation allows the correlation of human activities and vegetation changes. Our data collection and analysis provide insight into the collapse of Shijiahe culture in the middle Yangtze in China during the late Neolithic period. Phytolith and charcoal records from the excavation in Shijiahe city trace changes in rice production and ancient activities that were significantly affected by climate alteration.

The stratigraphic records of archaeological sites, AMS C-14 dating, and micropaleontological analysis indicate that the vegetation and climate evolution showed explainable change during late Qujialing-Shijiahe cultural period. Our observations and conclusions are comparable with other researchers’ records and show response to local ancient environmental and climate changes.

(1) During the late Qujialing-Shijiahe cultural stage (c, 5000–4000 cal BP), affected by the weakened East Asia Monsoon and Holocene Event 3, the climate experienced two different stages: from wet-warm (Qujialing- early Shijiahe culture) to a cold-dry period (middle-late Shijiahe culture). At that time, vegetation included forests, grass, and wet lands in this district. There was also undergrowth of the forest grasses including Panicoid, Choridoid, and Arundinoideae (Qujialing- early Shijiahe culture). In the middle-late Shijiahe culture stage, the climate became more dry and cold, the forest became gradually atrophic, the water in water-covered areas dried, and the vegetation became dominated by Pooideae, Festucoid and Bambusoid.

(2) In the late Qujialing to the early Shijiahe culture, favorable weather conditions allowed the robust development of agriculture. At that time, land clearing was widely performed by burning the native vegetation for the cultivation of large fields to produce crops. The land was reclaimed for rice cultivation to meet the needs of a significant increase in population size for the ancient city. This development was stopped by the reduction of water supply. In the middle of the Shijiahe culture, the climate became dry and cold, the available water retreated, and these changes resulted in the mass migration of the population. During the late Shijiahe culture, the dry and cold climate caused the water level to continue to fall. At the c, 4200 cal BP event, severe drought eroded the economic foundation of rice-cultivation, as indicated by the sharp decrease in rice phytolith concentration records. Finally, people were forced to abandon the ancient city, leading to the collapse of Shijiahe culture.

The continued excavation of archaeological sites can provide the opportunity to study the entire ancient city that was the center of Shijiahe culture. In addition, the ability to distinguish phytoliths for cultivated wild rice and millet can provide insight into the formation and evolution of the agricultural economy in the middle reach of the Yangtze River.
